# Applied Research in Low-Income Countries: Why and How?

**DOI:** 10.3389/frma.2019.00003

**Published:** 2019-11-14

**Authors:** Krishna Prasad Acharya, Santosh Pathak

**Affiliations:** ^1^Ministry of Land Management, Agriculture and Co-operative, Pokhara, Nepal; ^2^Department of Agricultural Economics and Agribusiness, Louisiana State University, Baton Rouge, LA, United States

**Keywords:** research, development, investment, low-income countries, economy

## Abstract

Research and development (R&D) offer promising clues to address a wide range of socioeconomic problems through the development of new products and services or often by improving the existing ones. High-income countries (HICs) have realized the worth of R&D and invested tremendously in that sector; however, resource-poor low-income countries (LICs) are still far behind in realizing the potential benefit that R&D could offer for economic growth and national development. Even if some LICs have a positive outlook towards the R&D sector, the trend of emulating works from HICs to solve local or regional issues have most often yielded counterproductive results. LICs are suggested primarily to focus on applied research by incorporating their socioeconomic and cultural aspects to solve their everyday problems whose investigation is often ignored in research-intensive nations. Moreover, applied research in LICs offers the potential to provide low-cost and innovative solutions to local and regional problems with global implications.

## Introduction

Research and Development, often referred to as R&D, constitutes activities that are aimed towards devising new services or products, and also improving existing products or services (Kenton, 2019). R&D generates new ideas and/or skills, thereby improving peoples' lives and the society in which they live. Therefore, R&D serves as an important means to address and overcome a wide range of socioeconomic and environmental problems.

Good research drives most of the advancements across all scientific disciplines. How do we know if climate change is real? We need to conduct research: plot long-run temperature, rainfall, and carbon emissions and analyze them to determine any significant trends that might be of concern. How do we know which medications will help us feel better when we are sick? We need to conduct research, perhaps ask people to participate in double-blind trials for new medications. How do we know which fertilizer best helps a plant grow? We need to conduct research, maybe conduct randomized controlled trials under various environmental settings. The medications that we take, fertilizers that we apply in fields and even gadgets, which have become integral to our lives, were part of the investigational program in the past and we only use them because researchers have examined them and determined that they are effective and helpful for our overall betterment. R&D necessitates resource allocation in advance; however, the resulting innovations serve to reduce the costs through more efficient production processes or the product itself (Kenton, 2019).

Scientific research is a pre-requisite for human and societal development. There is a strong correlation between the level of advancement of scientific research and the standard of living (Badr, 2018). Results from careful research can be utilized to create wealth, increase the nation's worth, and boost the socioeconomic and political situation of the country. Innovation through quality research and subsequent patent rights have an additive effect on a nation's wealth and positive ripple effects on the economy. For example, China succeeded in lifting its 700 million people by domestic innovations and new start-up businesses (NDRC, 2016; Trivedi, 2018). Similarly, South Korea and Israel also boosted its economy through intensive R&D and subsequent integration into the global market. South Korea presents a vivid example of how a country, comparable with other poorer countries of Asia and Africa in 1960s, transcended to a trillion-dollar economy in 2004 by integrating R&D into their national strategy and spending more than 4% of gross domestic product (GDP) annually on R&D, with the majority being on applied research sector (Reddy, 2011). Israel is also exemplary in how a country primarily occupied by arid land succeeded to become one of the largest exporters of agricultural commodities through innovation following uninterruptible research.

As of 2019, global spending on R&D has reached $1.7 trillion. Interestingly, only about 10 countries account for 80% of global spending (UIS, 2019). North America and western Europe are homes to most research (46.1%) followed by east Asia and the Pacific (40.6%). The least share (0.1%) of global research is constituted by central Asia followed by sub-Saharan Africa (0.8%). According to UIS (2019), the global average for R&D spending is 1.7% of GDP; however, the variation is very high. The variation is due to the influence on research goals arising from field specialization, social outlooks, cultural factors and philosophies in a given nation (Rahman, 1975). Investment in R&D serves as “bedrock for development” and is vital for the development of any nation in this era of globalization and increased competitiveness. The importance of R&D is amplified by the fact that every $1 invested in R&D generates around $2 in return (UIS, 2019). While the rates of return vary, R&D nonetheless serves as an important driver of economic growth and development of a nation.

With increasing competitiveness in the face of globalization, no nation can now aspire to progress without effectively conducting and utilizing results from scientific research which is the ultimate basis of national development in the form of knowledge-capital, human resource, economic growth, improved standard of living and environmental sustainability (Khan et al., 2007).

## Case of Low-Income Countries (LICs)

According to the World Bank, countries with gross national income per capita below $955 are LICs and include 33 countries (World Bank, 2016; Table 1). LICs have limited resources with most of them having a GDP size of < $500 billion and per capita GDP < $2,000 (CIA, 2019). LICs are mostly located in the southern hemisphere of the globe. While most countries in the northern hemisphere, high-income countries (HICs), have outpaced issues such as poverty and underdevelopment long ago, their counterparts in the south are still stricken with domestic conflict, poverty, malnutrition and food crisis leaving them far behind in terms of cherishing life amenities and modern infrastructural development. Keeping aside many factors influencing the success of HICs, one of them is that HICs were able to draw clues and trace a path to rapid development through timely and careful research and its subsequent development. HICs including the United States, Japan, and Great Britain, besides prospering themselves, inspired many other nations on how to identify the problems and tackle them through demand-driven research, ultimately benefiting citizens and leaving some spillovers around the globe. Today, if we look carefully at the way of living, infrastructures, ongoing innovation, national policies regarding both present and future goals and the like, we can feel that there are many small globes within our globe. Just standing somewhere in the United States, Germany, or Japan and conversely standing in Afghanistan, Somalia, or even Nepal can give a “big picture” of the vast disparity resembling completely different globes across different continents.

**Table 1:** List of LICs, MICs, and HICs by GDP per capita and R&D spending.

**Country**	**GDP per capita (US $)**	**Researchers per million population (in FTE^**†**^)**	**% of GDP spending on R&D**
**LICs**			
Afghanistan	586	–	–
Benin	830	–	–
Burkina Faso	671	47.6	0.67
Burundi	320	–	0.10
Central African Republic	418	–	–
Chad	670	58.3	0.32
Comoros	797	–	–
Democratic Republic of Congo	458	10.6	0.41
Ethiopia	548	45.0	0.60
The Gambia	501	33.6	0.10
Guinea	893	–	–
Guinea-Bissau	614	–	–
Haiti	766	–	–
Liberia	456	–	–
Madagascar	450	30.6	0.01
Malawi	338	48.3	–
Mali	825	32.8	0.29
Mozambique	416	41.5	0.34
Nepal	835	61.0	0.30
Niger	378	–	–
Rwanda	748	12.3	–
Senegal	1,033	549.3	0.75
Sierra Leone	499	–	–
Tajikistan	801	–	0.12
Togo	617	38.3	0.27
Uganda	604	26.5	0.17
Zimbabwe	1,080	88.7	–
**MICs**			
India	1,940	216.2	0.62
China	8,827	1234.8	2.13
**HICs**			
USA	59,532	4256.3	2.80
Japan	38,428	5304.9	3.20
South Korea	29,743	7514.4	4.55
Israel	40,560	8250.5	4.20

† *FTE represents full-time equivalent. GDP denotes gross domestic product. LICs, MICs, and HICs denote low-income countries, middle-income countries and high-income countries, respectively. As of 2017, the average R&D spending (% of GDP) in LICs, MICs, HICs, and global scale is 0.33, 1.12, 2.33, and 1.68%, respectively. Similarly, the number of researchers (FTE) per million inhabitants in LICs, MICs, HICs, and global scale is 149.4, 642.8, 4063.6, and 1162.7, respectively (Source: World Bank, 2017; UIS, 2019)*.

Among several other limitations, LICs have limited human development and limited expenditure in areas of education and scientific research (Helmy et al., 2016). The economy of LICs is primarily sustained by the agriculture and livestock sector. Around two-thirds of the population in these countries are under abject poverty and have pressing issues on education, health, and agriculture sector. Due in part from being unable to identify the cause of a plethora of problems that people face daily, LICs have still been lagging far behind to discover the most promising clue to what they are facing up with. Moreover, it can be said that LICs could not outpace their own previous self through timely research and development to embark on the journey of national development and the enhanced lifestyle of their citizens.

This opinion paper focuses on what LICs should consider in making their research different from those in HICs that are home to cutting edge and advanced research. We reviewed both academic and gray literature. Our paper has two main objectives: (i) identify problems of R&D in LICs, and (ii) weigh basic and applied research options for uplifting LICs out of socioeconomic problems that are specific to their land.

## Problems of R&D In LICs

LICs account for ~85% of the global disease burden with the majority of the population fighting against poverty-related malnutrition, infectious diseases (both airborne and waterborne), hunger, and environmental brunt like climate change, famine, water scarcity, and deforestation on a day-to-day basis (Batterman et al., 2009; Thomas, 2015). As a result, R&D is not under a government priority in LICs with investment being <1% of their GDP (Gaillard, 2010). This is unsurprising due to three reasons: First, LICs are still struggling to meet the necessities of food, clothing, and shelter of their citizens. This leaves the government with only a few surplus resources to invest in R&D. Furthermore, LICs typically finance most of their research with public funds, unlike HICs where the business sector funds most research activities. This fosters stronger budget austerity, making it more important to understand the effect of R&D budget allocation decisions (Gonzalez-Brambila et al., 2016). Research requires substantial financial investment over a protracted period. Some pioneer research might take up to a decade or even more to get meaningful results, while some other cutting-edge research after laboratory experimentation needs validation in the field condition. All of these processes demand perseverance and continuous financial commitment over a prolonged period that is difficult to secure in LICs.

Second, with more important social issues, political parties and bureaucrats in LICs believe that research is a sack into which money is poured and from which nothing of apparent value is reaped. They also perceive R&D as a waste of limited resources. This preconceived notion of political personnel and bureaucrats deters from making a proper budget allocation in the R&D sector. Instead, they focus on immediate needs having practical values: eradication of hunger, control of infectious and debilitating diseases, decrease in the unemployment rate, and raising the quality of life of their citizens, but in a conventional way. In other words, LICs are more focused on those issues that have immediate results to society and the economy as a whole. For example, the national campaign for vitamin A and polio vaccination, where simple intervention and low investment would have a greater and immediate impact saving millions of children from potential danger. Investment in such areas might seem rational over spending on R&D in the short term for them. The process of R&D is severely constrained by a small budget allocation from lack of knowledge and ignorance of that part.

Third, despite some research efforts, poor implementation of research findings is another pressing issue for LICs as a result of which research findings are not clearly linked with visible output (Hoekman et al., 2003). Besides that, some research fails to address the local culture, human rights issues, language policy, and local environment and thus is not translated into applicable outcomes. In agriculture, there are several instances where local agribusinesses bypass local science and technology (S&T) systems and rely on foreign technologies as a response to new innovation elsewhere thereby leading to loss of inherent profit potential (Keskin et al., 2008).

Insufficient research translates into data and knowledge gaps which are major constraints to future well-being and furthering development. An insufficient amount of quality research is the major impediment to growth, development, and advancement. So, raising awareness on the importance of R&D and a positive outlook towards its promising nature are very necessary (Kirigia and Barry, 2008).

## Reasons Behind Low Scientific Productivity in LICs

LICs face some of the world's toughest challenges, as stated earlier, which can be tackled through applied research based on their own context. The dilemma for LICs persists in part from inconsistencies in policies and the limited national budget for research activities to address a country's short-term and long-term concerns effectively. In contrast, the investment in basic and applied research has brought enormous benefits to the HICs, while LICs still lack a strategic approach in this regard (Khan et al., 2007). The conditions under which research is conducted in LICs are not without doubt and do not encourage engagement and continuity in research activity. Several factors are associated with declining scientific productivity in LICs. Some of them include restricted access to research grants, undersized laboratory infrastructures, inadequate budgets, limited equipment and reagents, and lack of professional security for scientists (Ciocca and Delgado, 2017).

Similarly, LICs lack a long-term vision with the consideration that science is a driver of the economy, which is indispensable for productive research and scientific development. When research is less emphasized due to political and economic instability, national industries lack incentives to produce goods and services at home, thus increasing imports of equipment and supplies from outside (Ciocca and Delgado, 2017). Moreover, societies in LICs also expect new scientific and technological breakthroughs to come from HICs instead of their own scientists. Such practices are widely prevalent in LICs that deter innovation and demotivate national scientists.

According to Ciocca and Delgado (2017), researchers' remuneration does not correspond to their education, knowledge, and societal contribution in LICs. They further assert that research enthusiasts in the LICs face with highly restricted, competitive, and poorly funded research environment. For example, most medical doctors find it sustainable to pursue clinical practice instead of engagement in research activities. Furthermore, researching in South Asia, Africa, or even Central America demands that factors such as cultural sensitivity, and resource limitations are appropriately addressed, besides patience and creativity (de Baessa, 2008).

The contributions of science to society are well evident through medical discoveries such as antibiotics and technological development including computers and smart-phones (Ciocca and Delgado, 2017). Therefore, HICs spend a substantial portion of their budgets on research. In contrast, research is considered as a minor activity and less prioritized in LICs. Hence, countries with insignificant research activities depend on nations with a robust research culture and act as the supplier of raw materials. Following Ciocca and Delgado (2017), the decline in scientific productivity results not from the shortfall of creativity in LICs but from the failure of leadership to create an appropriate research environment. Furthermore, there are no well-defined rules for distributing limited research funds. The limited research funds are mostly distributed based on political connections (Ciocca and Delgado, 2017) and further aggravated by economic instability, thereby resulting in a negative impact on innovation (Schot and Steinmueller, 2018). Despite the commitment of some of the LICs, the development of innovation capacity remains poor in most countries due in part to both external and internal brain drain. Both external and internal brain drains have been prominent due to modeling public financed science and technology systems based on advanced country institutions instead of their own. This results in a wasteful built-up of human and capital resources that are unable to contribute to the advancement of local socioeconomic systems (Clark and Chataway, 2009).

## Which is Appropriate For LICs: Basic or Applied Research?

Research, as previously cited, is the pursuit of knowledge while development uses the results of research to develop “new products, methods, and means of production” (Niiniluoto, 1993). There are two types of research: basic and applied. Basic research, applied research, and development are closely interlinked in the form of the R&D cycle (Riazuddin, 2007). They are not only the source of new knowledge and understanding but also of product/s and process/es. Basic research paves the way to applied research, which accelerates the development process and conversely stimulates new pathways for basic research to generate deeper fundamental understanding (Khan et al., 2007). However, advances in basic research do not always arise from advances in R&D. As mentioned in Khan et al. (2007), “the R&D cycle, thus, works constantly to expand the frontiers of knowledge, as well as, to enhance the pace of development.” Basic research emphasizes “big picture” topic such as expanding the scientific knowledge base about a specific subject matter (Cherry, 2018). However, applied research focuses on solving a specific, practical problem of individuals or societies. Unlike basic research, applied research is concerned with resolving common problems that affect life, work, health, and overall well-being (Cherry, 2018). Moreover, applied research prioritizes more on fixing particular problems that frequently affect people. However, different they might seem, basic and applied research are closely intertwined. Basic research often informs applied research; applied research often helps basic researchers refine their theories. Basic research is essentially curiosity-driven while applied research is problem-oriented and used for a mission for a specified period. After having realized the importance of research for the development of a knowledge-based economy, the LICs ought to prioritize their research activities and create a balance between basic and applied research with more priority to applied research at the beginning and gradual shift to the basic research sector (Khan et al., 2007). There are several reasons behind the urge to focus on applied research in LICs at the existing condition of backwardness and the struggle of people to meet basic life amenities.

Applied research begins with the identification of a real-world problem. Furthermore, applied researchers discern the cause of the problem and investigate alternative solutions for that problem (Cherry, 2018). While the primary objective of applied research is solving real-world problems, it also adds to the knowledge base about the evolution and consequences of different problems. Such information serves as a useful future reference for related problems. The very little money available for research in LICs can be justified when it gets attached to daily lives with implications (Cherry, 2018). There is no doubt that basic and applied research in LICs are guided by universal scientific principles, yet there are some differences that set apart.

According to Russell and Galina (1998) issues such as remoteness from standard scientific practices, demand for advancement of native scientific capacity, lack of extensive research community, need for enhancing outreach and extension coupled with the need to develop local “new” science to resolve critical local problems suggest that LICs possess a unique set of conditions compared to that found in HICs. Moreover, problems unique to particular LICs will remain ignored by the global scientific community until they too are affected, as was seen in the case of the Ebola epidemic (Omoleke et al., 2016).

Applied R&D bears the perks of both rapid results in local conditions and generates employment to residents of a particular location. LICs should focus, at first, on groundwork applied research on building capabilities or low level of innovation until the country has adequate funds to invest in novel research. Research in LICs, however, should adhere to international quality while addressing the needs and priorities of a particular society/nation by taking into account the social, political, economic, and other external contextual domains. If done so, LICs can do better than others and excel in comparison to their counterparts from the developed world taking benefit of their own geographical and cultural diversity, and their own resources. In addition to that, they can develop cutting edge technologies to address global issues despite resource crunch (Harris et al., 2017). Regarding the economic burden of R&D, public expenditure is productive up to some extent; however, once the growth phase takes wing it is desirable to shift to R&D mostly driven by the private sector. It is because the escalated pace of R&D in HICs also mostly arises from the private or business sector driven research activities.

## Some Examples of Promising Applied Research From LICs

Despite several hurdles on their way, both in the past and at present, many countries outside HICs have succeeded to generate findings having a significant impact on the life of millions. While developed nations invest a lot in forest conservation, the ‘community forest' approach in Nepal provides a striking example of low-cost forest conservation and sustainable management options. System of Rice Intensification (SRI), a system demanding less water but increasing yields developed from Madagascar, offers an easy to adopt and profitable alternative for rice farmers (de Laulanié, 2011). Ophthalmic research in Tilganga Institute of Ophthalmology in Nepal is exemplary in the whole world for its low-cost lens restoring sights of millions of poor patients (Moran et al., 1997; Allan, 2000). Similarly, Novel Prize-winning ‘Grameen Bank' is also the outcome of a research project to design the credit delivery system in a way to provide banking services to the rural poors (Dowla, 2006). This project is exemplary in proving that loans are better than charity to fight poverty. Now, the concept of microfinance is popular across many LICs to combat poverty and promoting the livelihood of rural poors. National Innovation Center in Nepal is also working to find innovative solutions to different local problems. One of the interesting successes is that they invented low-cost machines that help chase invading monkeys from farmers' fields. Simple it may seem, but it serves to the huge benefit of farmers who have not been able to save their crops from invading monkeys. Similarly, their research on drone-based drug delivery system, if successful, will prove phenomenal in supplying medicines to the people in remote hills of Nepal. Middle-income countries (MICs) like Brazil, China, Russia, and India, which traditionally played a secondary role in the global innovation realm, now provide good examples from sectors like electronics, communication and information technology on how to develop and sufficiently build-up own innovation capabilities (Mathews, 2006).

Notably, some uninvolved applied research might have huge implications. A simple understanding of the soil bacteria of the particular locality could later be applied for further research on the suitability of crops in the given area. Recombinant DNA technology is useful to modify plants genetically in order to increase crop yield and/or improve nutritional content. This could solve LICs' problems: chronic malnutrition and food insecurity. Applied research also addresses challenges that represent a real threat to human existence such as climate change. Good manufacturing practices and thermal processing in food industries offer options to make available a wide range of food choices. Applied research also extends its hands to solve marketing problems and take advantage of marketing opportunities. Issues such as food waste can be tackled only through applied research. Wind erosion control, pitched roads from plastic materials and water harvesting from foggy hills were possible through careful applied research. These research were meant to solve problems specific to a particular land and later adopted across boundaries, often through small modifications. Applied research, which appears so simple today, can provide valuable insights and additional benefits to our lives and the overall human society.

## Further Options For Enhancing R&D In LICs

People working in fundamental fields serve as ‘idea man' to applied colleagues (Moravcsik, 1988). Fundamental science of today will become applied science of tomorrow (Moravesik, 1964). Applied research can be best carried out if the researchers have constant access to people working in fundamental research (Moravesik, 1964). However, the problem with LICs is that they do not have many researchers on a specific topic. Therefore, LICs should focus on collaboration outside the border. Such collaboration can be in the form of retired colleagues or professors on sabbatical from HICs traveling to offer short-term intensive training courses; cross-cultural research; partnership work with international universities; and, donating journals to libraries in LICs (Moravesik, 1964). Even within the national boundary, promising innovation rarely happens in isolation; cooperation at an early stage among academia, the government and the private sector at a local level and even regional level is deemed necessary. Innovation is useless on its own without the market. The long-term goal should be on advancing to the global market ensuring autonomy and creativity of the private sector. LICs should prioritize job creation and diversify their economies (UNIDO, 2019).

When economic 5-year plans are set up, a corresponding 50-year plan should also be considered. Moreover, part of this must be the fostering of pure research, perhaps on a small scale starting today. The young scientist of today should be seen as the head of schools tomorrow whose works shall inspire the rest of others to conduct works comparable with advanced countries (Moravesik, 1964).

LICs need to focus on building firms' capabilities or low-level innovation until a country has adequate funds to invest in novel scientific R&D. Secondarily, they can benefit from social innovation taking into account their own cultural and social diversity. LICs can identify potentialities and utilize the resources to the most profitable enterprises in their own context. LICs can translate the findings of pioneer and basic research from HICs in their land by taking benefit of comparative advantage and utilizing local resources at relatively low cost to generate profitable output. Unlike in HICs, LICs should equally consider economic status, moral values, beliefs, culture and ultimately people's lives. In addition to being practical and problem solving, research should take into account the way that the majority of the impoverished population in LICs are benefitted.

LICs can also take the reference of basic research and translate the benefits of intervention in heterogeneous settings through operational research. This can be phenomenal to assess the feasibility of new strategies in specific settings and advocate for policy change (Zachariah et al., 2009). More specifically, research using less than a worldwide method in the health sector have been severely critiqued; however, investigators should be allowed to use less than worldwide best method when it is ethically appropriate and bears the potential to provide sufficient benefits for the host communities (Wendler et al., 2004). Taking an example of cancer in resource-constrained countries, strategies like deploying primary and secondary caregivers, using off-patent drugs, and applying regional/global mechanisms reduce the cost of prevention and treatment along with increased access to health services and strengthened health system (Farmer et al., 2010). Besides that, research in non-communicable diseases, cancer for example, in LICs will benefit not only the host countries but also could yield clues to a low-cost solution to the burden of disease worldwide (Hofman et al., 2006).

Besides adequately provisioning for R&D, LICs need to translate the outcomes of research into policy practice. Uptake of research findings can be promoted into policy and extended where relevant. If evidence-based policymaking is promoted, hopefully, it will generate better outcomes for its citizens. Besides the policy instrument, the nation must also provide incentives to scientists who make breakthroughs in science and technology; develop international relations in social, economic, cultural, and scientific spheres; modify school curriculum with a higher emphasis on creativity and spontaneity of children; relax portion of corporate tax for those developing innovative product and production process; and, special focus to encourage local organizations to promote innovation activities (Khayyat and Lee, 2015). Above all, funding research with a sense of obligation or to meet some targets won't do all good; the government along with the people should believe in its inherent importance.

Research can sustain in an environment that prioritizes, supports, and appreciates its importance. An environment of political or cultural intolerance to research has a stifling effect on research efforts. Governments in LICs can establish two types of institutions depending upon their needs and availability of resources: research universities/institutions for long-term strategic research and local institutes for addressing short-term or day-to-day problems. The first type of institution can set forth distant goals, identify resources and pave the way to achieving such goals. The latter one can work to deal with smaller, yet important problems like controlling diseases, improving market mechanisms, etc. that impact daily lives. If both types of institutions are carefully nurtured, it is no doubt that they can come up with pioneer ideas and breakthrough innovations.

Research University is pivotal in the era of globalization to educate a new generation for technological and intellectual leadership in a range of disciplines with a commitment to create and disseminate knowledge. Regional university such as South Asian University^1^ and global university such as the UN University of Tokyo^2^ offer striking examples to furthering the need for research universities. They can serve to be a go-to think tank for impartial research on the pressing global problems of human survival, hunger, poverty, climate change, and welfare. However, it is very difficult for a LIC to have its own research university; both economy and manpower delimit this prospect. Regional alliance in academia can be a milestone to cumulate strength in specific fields and participate in global science. Inter-linkage of academic institutions with the global academic system can also be promising to undertake research of both national and regional importance (Altbach, 2009, 2013).

The country can increase the proportion of fundamental/frontier research with the increasing success of applied research and the provision of adequate resources and tools to do so. Strong emphasis should be placed on applicability and representativeness of research, capacity building, self-reliance, and sustainability. Instead of investing in hi-tech R&D, LICs should emphasize the low-cost approach to innovation with immediate values to existing local issues. This can later be exemplary in solving global issues by simple modification. Above all, it is important to adjust R&D activities to address the pressing needs of a society/nation rather than trailing wanderlust with international research wagon.

Many brilliant young minds emigrate from LICs to HICs, where both the growth potential and the likelihood of being successful are higher. Despite this, LICs have many passionate young graduates willing to explore their career in science and further societal well-being. It is therefore the government's responsibility to improve the appalling situation of science for the sake of aspiring graduates that portray the intellectual and economic future of their countries (Ciocca and Delgado, 2017). If LICs succeed to provide appropriate space to scientists and researchers trained in advanced countries, they can still duly benefit from their citizens dwelling in HICs. However, for that to turn into reality, the nation must be open to consider their potentials and deploy them through meritocracy to solve societal deep-rooted problems from proper research.

Above all, LICs should link up their citizens and scientists via science education and the active participation of researchers in problems within the community (Valenzuela, 2014). The government in LICs must learn from the example of HICs and invest in long-term research goals that are stable even during the shift from one political party to another (Ciocca and Delgado, 2017). Moreover, the path to the scientific and technological development of a country is cumulative, interlinked, and insistent (da Silva, 2016). Hence, a strong commitment to research is inevitable to enhance the quality of life in a society. There is also the need to stimulate social entrepreneurship and bottom-up local innovation in lieu of traditional aid and technology transfer as the ability to innovate is critical to the growth and future performance of firms (Khayyat and Lee, 2015; Wieczorek, 2018).

## Strengths of R&D In LICs

Moravcsik termed “local new science” for local scientific topics and stressed on carrying out research more oriented on the same (Moravcsik, 1985). It often refers to applied Research Topics focusing on solving problems that are either no longer of concern to HICs or that have a specific geographical, cultural, political, or philosophical context in some or all of the LICs as mentioned by Russell and Galina (1998). Generally, it is assumed that frontiers of science will benefit only the richer nations; however, in reality, resource-poor settings drive innovation demanding inexpensive product design requiring less infrastructure and are easy to use (Elias, 2006).

Some of the issues LICs face are country-specific, but many will be systemic. Consequently, overcoming the problems requires the involvement of both domestic governments and the wider economic community including international financial institutions, donors and academics (Bevan, 2012). This will help form regional alliances to deal with common problems and will subsequently contribute to the progress of the overall region. Extra-regional collaborations can help nations address challenges that extend beyond their region while intra-regional scholarly collaborations help address common regional challenges in areas of agriculture, medicine, and infrastructure. International collaborations can yield highly significant benefits among LICs by enabling them to leverage and complement the resources of countries with a solid research base (Elsevier, 2019).

LICs also offer a great venue for common problems at a very low cost due in part from the low cost of labor, inputs, and less stringent legal procedures. LICs can potentially provide a venue to innovate hybrid technology by using pieces of evidence from established research.

According to Clarivate Analytics, between 2006 and 2016, the number of highly cited papers featuring Africa-based authors increased by 400% (Web of Science, 2017). This spreads rays of hopes to researchers working in resource-constrained environments. Furthermore, the Open Access movement in scholarly publications now provides access to the most recent research more than ever before (Tennant et al., 2016). This might prove to be phenomenal to disseminate local knowledge, innovations, and bridge the north-south knowledge gap (Chan and Costa, 2005).

## Conclusion

R&D is an indispensable part of the invention and scaling up of products and services. R&D is also essential to puzzle out different phenomenon in and around us for enhancing the quality of life. While HICs have used R&D to significantly mobilize their resources and foster economic development, LICs are still struggling in that regard. This era of globalization and increasing competitiveness demands that LICs also utilize the potential that R&D offers to ensure sustained economic growth and enhance the way of living. Of the two research options, basic and applied, LICs could potentially benefit from the latter option at a beginning phase as applied research is concerned with solving specific problems affecting people in the particular vicinity at a given time. Applied research could also help LICs to deal with their poverty, diseases, malnutrition, conflict, famine, and even environmental degradation while providing the possibility of extending promising results in heterogeneous settings across the boundaries. The findings of applied research, not undermining basic research, serve as a tool for evidence-based policymaking and most likely provide better outcomes to citizens in a way that their lifestyle is positively impacted. In conditions of existing resources and financial limitations in LICs, regional alliance and global partnership are expected to tackle common problems and harness the benefits of collaboration in a more efficient and feasible manner.

## Author Contributions

KA conceived the idea and wrote the initial manuscript. SP extensively revised the paper. KA and SP finalized and approved for publication.

### Conflict of Interest

The authors declare that the research was conducted in the absence of any commercial or financial relationships that could be construed as a potential conflict of interest.

## Footnotes

^1^South Asian University, New Delhi. Available online at: www.sau.int.

^2^United Nations University, Tokyo. Available online at: www.unu.edu.
