# Corrigendum: Applied Research in Low-Income Countries: Why and How?

**DOI:** 10.3389/frma.2021.667663

**Published:** 2021-06-01

**Authors:** Krishna Prasad Acharya, Santosh Pathak

**Affiliations:** ^1^Ministry of Land Management, Agriculture and Co-operative, Pokhara, Nepal; ^2^Department of Agricultural Economics and Agribusiness, Louisiana State University, Baton Rouge, LA, United States

**Keywords:** research, development, investment, low-income countries, economy

In the second paragraph of the **Introduction** of the original article, there was an incomplete sentence with a missing citation and overlapping text stating that “Although resources must be allocated in advance to R&D, the innovations gained through the research can actually work to reduce costs through more efficient production processes or the product itself.” This sentence should read, “R&D necessitates resource allocation in advance; however, the resulting innovations serve to reduce the costs through more efficient production processes or the product itself (Kenton, 2019).” Correction to the overlapped texts has been made along with addition of a citation to the fifth paragraph of the **Introduction** and should read: With increasing competitiveness in the face of globalization, no nation can now aspire to progress without effectively conducting and utilizing results from scientific research which is the ultimate basis of national development in the form of knowledge-capital, human resource, economic growth, improved standard of living and environmental sustainability (Khan et al., 2007). Corrections have been made with overlapping texts and misisng citations to the first, second, third, and fourth paragraphs of **Reasons Behind Low Scientific Productivity in LICs** and missing citations such as Khan et al. (2007), Ciocca and Delgado (2017), and Clark and Chataway (2009) are added and overlapped text are corrected, which should read:

LICs face some of the world's toughest challenges, as stated earlier, which can be tackled through applied research based on their own context. The dilemma for LICs persists in part from inconsistencies in policies and the limited national budget for research activities to address a country's short-term and long-term concerns effectively. In contrast, the investment in basic and applied research has brought enormous benefits to the HICs, while LICs still lack a strategic approach in this regard (Khan et al., 2007). The conditions under which research is conducted in LICs are not without doubt and do not encourage engagement and continuity in research activity. Several factors are associated with declining scientific productivity in LICs. Some of them include restricted access to research grants, undersized laboratory infrastructures, inadequate budgets, limited equipment and reagents, and lack of professional security for scientists (Ciocca and Delgado, 2017).

Similarly, LICs lack a long-term vision with the consideration that science is a driver of the economy, which is indispensable for productive research and scientific development. When research is less emphasized due to political and economic instability, national industries lack incentives to produce goods and services at home, thus increasing imports of equipment and supplies from outside (Ciocca and Delgado, 2017). Moreover, societies in LICs also expect new scientific and technological breakthroughs to come from HICs instead of their own scientists. Such practices are widely prevalent in LICs that deter innovation and demotivate national scientists.

According to Ciocca and Delgado (2017), researchers' remuneration does not correspond to their education, knowledge, and societal contribution in LICs. They further assert that research enthusiasts in the LICs face with highly restricted, competitive, and poorly funded research environment. For example, most medical doctors find it sustainable to pursue clinical practice instead of engagement in research activities. Furthermore, researching in South Asia, Africa, or even Central America demands that factors such as cultural sensitivity, and resource limitations are appropriately addressed, besides patience and creativity (de Baessa, 2008).

The contributions of science to society are well evident through medical discoveries such as antibiotics and technological development including computers and smart-phones (Ciocca and Delgado, 2017). Therefore, HICs spend a substantial portion of their budgets on research. In contrast, research is considered as a minor activity and less prioritized in LICs. Hence, countries with insignificant research activities depend on nations with a robust research culture and act as the supplier of raw materials. Following Ciocca and Delgado (2017), the decline in scientific productivity results not from the shortfall of creativity in LICs but from the failure of leadership to create an appropriate research environment. Furthermore, there are no well-defined rules for distributing limited research funds. The limited research funds are mostly distributed based on political connections (Ciocca and Delgado, 2017) and further aggravated by economic instability, thereby resulting in a negative impact on innovation (Schot and Steinmueller, 2018). Despite the commitment of some of the LICs, the development of innovation capacity remains poor in most countries due in part to both external and internal brain drain. Both external and internal brain drains have been prominent due to modeling public financed science and technology systems based on advanced country institutions instead of their own. This results in a wasteful built-up of human and capital resources that are unable to contribute to the advancement of local socioeconomic systems (Clark and Chataway, 2009).

The first and second paragraphs of **Which is appropriate for LICs: Basic or Applied Research?** were incomplete with missing citations for Niiniluoto (1993), Khan et al. (2007), and Russell and Galina (1998) and some overlapped texts. The overlapped texts have been corrected and those paragraphs should read:

Research, as previously cited, is the pursuit of knowledge while development uses the results of research to develop “new products, methods, and means of production” (Niiniluoto, 1993). There are two types of research: basic and applied. Basic research, applied research, and development are closely interlinked in the form of the R&D cycle (Riazuddin, 2007). They are not only the source of new knowledge and understanding but also of product/s and process/es. Basic research paves the way to applied research, which accelerates the development process and conversely stimulates new pathways for basic research to generate deeper fundamental understanding (Khan et al., 2007). However, advances in basic research do not always arise from advances in R&D. As mentioned in Khan et al. (2007), “the R&D cycle, thus, works constantly to expand the frontiers of knowledge, as well as, to enhance the pace of development.” Basic research emphasizes “big picture” topic such as expanding the scientific knowledge base about a specific subject matter (Cherry, 2018). However, applied research focuses on solving a specific, practical problem of individuals or societies. Unlike basic research, applied research is concerned with resolving common problems that affect life, work, health, and overall well-being (Cherry, 2018). Moreover, applied research prioritizes more on fixing particular problems that frequently affect people. However, different they might seem, basic and applied research are closely intertwined. Basic research often informs applied research; applied research often helps basic researchers refine their theories. Basic research is essentially curiosity-driven while applied research is problem-oriented and used for a mission for a specified period. After having realized the importance of research for the development of a knowledge-based economy, the LICs ought to prioritize their research activities and create a balance between basic and applied research with more priority to applied research at the beginning and gradual shift to the basic research sector (Khan et al., 2007). There are several reasons behind the urge to focus on applied research in LICs at the existing condition of backwardness and the struggle of people to meet basic life amenities.

Applied research begins with the identification of a real-world problem. Furthermore, applied researchers discern the cause of the problem and investigate alternative solutions for that problem (Cherry, 2018). While the primary objective of applied research is solving real-world problems, it also adds to the knowledge base about the evolution and consequences of different problems. Such information serves as a useful future reference for related problems. The very little money available for research in LICs can be justified when it gets attached to daily lives with implications (Cherry, 2018). There is no doubt that basic and applied research in LICs are guided by universal scientific principles, yet there are some differences that set apart.

According to Russell and Galina (1998) issues such as remoteness from standard scientific practices, demand for advancement of native scientific capacity, lack of extensive research community, need for enhancing outreach and extension coupled with the need to develop local “new” science to resolve critical local problems suggest that LICs possess a unique set of conditions compared to that found in HICs. Moreover, problems unique to particular LICs will remain ignored by the global scientific community until they too are affected, as was seen in the case of the Ebola epidemic (Omoleke et al., 2016).

Correspondingly, the 9th and 10th paragraphs of **Further options for enhancing R&D in LICs** had incomplete sentences, overlapped texts and a missing citation for Ciocca and Delgado (2017), which have been corrected and should read:

Many brilliant young minds emigrate from LICs to HICs, where both the growth potential and the likelihood of being successful are higher. Despite this, LICs have many passionate young graduates willing to explore their career in science and further societal well-being. It is therefore the government's responsibility to improve the appalling situation of science for the sake of aspiring graduates that portray the intellectual and economic future of their countries (Ciocca and Delgado, 2017). If LICs succeed to provide appropriate space to scientists and researchers trained in advanced countries, they can still duly benefit from their citizens dwelling in HICs. However, for that to turn into reality, the nation must be open to consider their potentials and deploy them through meritocracy to solve societal deep-rooted problems from proper research.

Above all, LICs should link up their citizens and scientists via science education and the active participation of researchers in problems within the community (Valenzuela, 2014). The government in LICs must learn from the example of HICs and invest in long-term research goals that are stable even during the shift from one political party to another (Ciocca and Delgado, 2017). Moreover, the path to the scientific and technological development of a country is cumulative, interlinked, and insistent (da Silva, 2016). Hence, a strong commitment to research is inevitable to enhance the quality of life in a society. There is also the need to stimulate social entrepreneurship and bottom-up local innovation in lieu of traditional aid and technology transfer as the ability to innovate is critical to the growth and future performance of firms (Khayyat and Lee, 2015; Wieczorek, 2018).

Russell and Galina (1998) was not cited in paragraph 1 of **Strengths of R&D in LICs**. It has now been added and should read: Moravcsik termed “local new science” for local scientific topics and stressed on carrying out research more oriented on the same (Moravcsik, 1985). It often refers to applied Research Topics focusing on solving problems that are either no longer of concern to HICs or that have a specific geographical, cultural, political, or philosophical context in some or all of the LICs as mentioned by Russell and Galina (1998). Generally, it is assumed that frontiers of science will benefit only the richer nations; however, in reality, resource-poor settings drive innovation demanding inexpensive product design requiring less infrastructure and are easy to use (Elias, 2006).

In paragraph 4 of **Strengths of R&D in LICs**, a reference was missing in a statement stating that “Furthermore, the Open Access movement in scholarly publications now provides access to the most recent research more than ever before.” This sentence should read, “Furthermore, the Open Access movement in scholarly publications now provides access to the most recent research more than ever before (Tennant et al., 2016).” Similarly, the first two sentences of **Conclusion** were inconclusive with overlapping texts stating, “R&D is not just about creating new products; it is also about strengthening existing products and services with additional features. Simply speaking, R&D is about striving for the betterment of our lives and understanding different phenomena around us.” This has been corrected and should read: “R&D is an indispensable part of the invention and scaling up of products and services. R&D is also essential to puzzle out different phenomenon in and around us for enhancing the quality of life.”

The authors found some overlapping texts and missing references in the original article, which have been corrected in this corrigendum. The authors sincerely apologize for the errors and omissions and state that this does not alter the conclusions of the article in any way. The article has been updated and the original published version has been included as supplementary material.
